# Critical Role of a Ferritin-Like Protein in the Control of *Listeria monocytogenes* Cell Envelope Structure and Stability under β-lactam Pressure

**DOI:** 10.1371/journal.pone.0077808

**Published:** 2013-10-24

**Authors:** Agata Krawczyk-Balska, Magdalena Lipiak

**Affiliations:** Department of Applied Microbiology, Faculty of Biology, University of Warsaw, Warsaw, Poland; INRA Clermont-Ferrand Research Center, France

## Abstract

The human pathogen *Listeria monocytogenes* is susceptible to the β-lactam antibiotics penicillin G and ampicillin, and these are the drugs of choice for the treatment of listerial infections. However, these antibiotics exert only a bacteriostatic effect on this bacterium and consequently, *L. monocytogenes* is regarded as β-lactam tolerant. It is widely accepted that the phenomenon of bacterial tolerance to β-lactams is due to the lack of adequate autolysin activity, but the mechanisms of *L. monocytogenes* tolerance to this class of antibiotics are poorly characterized. A ferritin-like protein (Fri) was recently identified as a mediator of β-lactam tolerance in *L. monocytogenes*, but its function in this process remains unknown. The present study was undertaken to improve our understanding of *L. monocytogenes* tolerance to β-lactams and to characterize the role of Fri in this phenomenon. A comparative physiological analysis of wild-type *L. monocytogenes* and a *fri* deletion mutant provided evidence of a multilevel mechanism controlling autolysin activity in cells grown under β-lactam pressure, which leads to a reduction in the level and/or activity of cell wall-associated autolysins. This is accompanied by increases in the amount of teichoic acids, cell wall thickness and cell envelope integrity of *L. monocytogenes* grown in the presence of penicillin G, and provides the basis for the innate β-lactam tolerance of this bacterium. Furthermore, this study revealed the inability of the *L. monocytogenes* *Δ* *fri* mutant to deplete autolysins from the cell wall, to adjust the content of teichoic acids and to maintain their D-alanylation at the correct level when treated with penicillin G, thus providing further evidence that Fri is involved in the control of *L. monocytogenes* cell envelope structure and stability under β-lactam pressure.

## Introduction

*Listeria monocytogenes* is a ubiquitous Gram-positive opportunistic pathogen that causes rare but severe disease in humans and animals. While listeriosis may occur in otherwise healthy individuals, those primarily at risk are immunocompromised patients, pregnant women, the very young and the elderly. Septicemia, meningitis and other infections of the central nervous system are commonly seen in patients with listeriosis. In at-risk groups, the mortality rate is 20–30%, even with antibiotic treatment, making listeriosis one of the most deadly bacterial infections [1], [2].

The treatment of choice for *L. monocytogenes* infections is a β-lactam antibiotic (penicillin G or ampicillin), alone or in combination with an aminoglycoside [3]. The general susceptibility of *L. monocytogenes* isolates to β-lactams is reflected by the low MIC (minimal inhibitory concentration) of these antibiotics. However, the MIC and MBC (minimal bactericidal concentration) values of β-lactam antibiotics against most isolates of *L. monocytogenes* are markedly different, and consequently this bacterium is regarded as tolerant to β-lactams [3], [4]. The tolerance of *L. monocytogenes* to β-lactams is one of the most important factors contributing to the not infrequent failure of antibiotic therapy against listeriosis. The mechanism underlying *L. monocytogenes* tolerance to β-lactams is largely unknown.

In other bacterial species, β-lactam tolerance is caused by reduced activity of murein (peptidoglycan) hydrolases, also referred to as autolysins. Peptidoglycan hydrolases are present in all bacteria synthesizing murein cell walls, and due to their ability to catalyze selective hydrolysis of covalent bonds in murein, they are involved in numerous cellular processes including cell growth, cell wall turnover, murein maturation, cell division, separation, differentiation and pathogenicity [5], [6]. These enzymes are also believed to promote bacterial cell lysis in response to reduced penicillin binding protein activity following treatment with β-lactams [7], [8], [9]. Five *L. monocytogenes* autolysins have been identified so far: Iap (Invasion associated protein), also known as CwhA (cell-wall hydrolase A) or p60 (60 kDa protein); MurA (muramidase A) also called NamA (N-acetylmuramidase A); Spl (p45); Ami and Auto [10], [11], [12], [13], [14], [15].

Since murein hydrolases play a crucial role in bacterial cell lysis, their activity is subject to tight control. In the case of Gram-positive bacteria, a complex control mechanism regulating autolysin activity was proposed in which the main role is played by teichoic acids (TA), which inhibit murein hydrolase activity depending on the presence or absence of protonated D-alanines [16]. TA are polyanionic polymers of Gram-positive bacteria, covalently bound to peptidoglycan in the cell wall. Different species have diverse requirements for TA, suggesting that this polymer may perform various functions. As major constituents of the surfaces of Gram-positive bacteria, TA influence a number of important biological processes, such as autolysis, the binding of cations and surface-associated proteins, cell adhesion, biofilm formation, coaggregation, resistance to antimicrobial agents such as cationic peptides, protein secretion, acid tolerance, virulence and stimulation of the host immune response [17], [18]. The modification of TA with D-alanine is highly conserved and it occurs in the cell wall compartment after TA biosynthesis is completed [17]. The *dltABCD* operon encodes the enzymes catalyzing this process, and is therefore responsible for modulating the net charge of teichoic acid polymers [19].

It was recently shown that Fri, a ferritin-like protein, plays a crucial role in the tolerance of *L. monocytogenes* to β-lactam antibiotics [20]. Fri of *L. monocytogenes* belongs to the Dps (*D*NA-binding *p*roteins from *s*tarved cells) family of proteins, which play an important role in counteracting the adverse effects of iron under aerobic conditions. Dps family proteins resemble ferritins and heme-containing ferritins (bacterioferritins) in their structure and function [21]. Thus, these proteins form cage-like polymers with iron oxidation/storage properties that serve two purposes. One is the removal of Fe(II) from the cytoplasm to prevent the potential generation of the highly toxic hydroxyl radical through the Fenton reaction: Fe(II) + H_2_O_2_→Fe(III) + OH^−^ + OH^•^. The other is to overcome the low solubility of Fe(III) by storing it in the protein internal cavity as ferric hydroxide micelles. Dps proteins use H_2_O_2_ as the physiological iron oxidant [22], [23], whereas ferritins employ molecular oxygen [24]. This characteristic enables Dps proteins to protect microorganisms from both Fe(II) and H_2_O_2_. There is significant variability in the type and number of ferritin-like proteins expressed in different bacterial species. *Escherichia coli* and *Salmonella enterica* possess two ferritins, one bacterioferritin and a Dps protein [25], [26], *Porphyromonas gingivalis* and *Campylobacter jejuni* contain one ferritin and a Dps protein [27], [28], while *Bacillus subtilis* and *Bacillus anthracis* contain two Dps proteins [29], [30]. This coexistence of multiple ferritin-like proteins within a single bacterium suggests that each protein plays a distinct physiological role, as exemplified in the case of *S. enterica* [26]. *L. monocytogenes* is unusual in that it possesses only a single Dps protein encoded by the *fri* gene [31].

The expression of *fri* in *L. monocytogenes* is subject to complex control. Transcription of the *fri* gene originates from three distinct promoters, one σ^B^-dependent and two σ^A^-dependent [32]. It is also known to be repressed by Fur and derepressed in a perR mutant background [33], [34], and to be upregulated under conditions of iron limitation, heat and cold shock [35], [36]. The ferritin-like protein of *L. monocytogenes* contributes to virulence and plays a role in protection against multiple stresses including oxidative, iron-starvation, cold- and heat-shock [32], [37], [38]. In addition, the deletion of *L. monocytogenes fri* was shown to abolish the innate tolerance of this bacterium to β-lactam antibiotics, which was accompanied by visible lysis of the cells [20], [39]. These observations led us to hypothesize that the role of Fri protein in the tolerance of *L. monocytogenes* to β-lactams might be associated with the regulation of bacterial cell envelope integrity.

This study was undertaken to pinpoint the role of Fri in the tolerance of *L. monocytogenes* to β-lactams. Fri was found to participate in the regulation of the components that are vital to the stability and function of the Gram-positive cell envelope. Under adverse conditions, such as β-lactam pressure, the activity of this protein is indispensable for the maintenance of *L. monocytogenes* cell envelope integrity. These findings also provide insight into the basis of the innate tolerance of this pathogen to β-lactams.

## Materials and Methods

### Bacterial strains, media and DNA techniques

*Escherichia coli* strain DH5α used in cloning experiments was grown in Luria-Bertani medium. The *L. monocytogenes* EGD wild-type strain and isogenic EGDΔ*fri* deletion mutant were grown in brain heart infusion (BHI, Oxoid) medium. When required, the media were supplemented with kanamycin (50 µg/ml). The EGDΔ*fri* deletion mutant [32] was a generous gift from Hanne Ingmer, Royal Veterinary and Agricultural University, Denmark. Plasmid pTCV-*lac* was a kind gift from Birgitte H. Kallipolitis, University of Southern Denmark, Denmark. The isolation of chromosomal and plasmid DNA, digestion of DNA with restriction enzymes, and PCR were performed according to standard protocols [40].

### Growth conditions

The *L. monocytogenes* strains used in all experiments were cultured as described previously [20]. Briefly, the strains were grown overnight in BHI medium at 37°C with shaking. Each culture was then diluted 1∶100 into fresh BHI broth with no additions or supplemented with 0.09 µg/ml penicillin G (Sigma-Aldrich), which equates to 0.75 of the MIC value. The prepared cultures were then incubated as described above until they attained an OD_600_ of ∼0.35, which corresponds to the mid-log phase of growth.

### Analysis of cell wall integrity

*L. monocytogenes* cultures were harvested by centrifugation and the cells resuspended in lysis buffer (25 mM phosphate buffer, pH 6.4) to an OD_600_ of 1.0. Hen egg lysozyme (Sigma-Aldrich) was then added to a final concentration of 10 µg/ml and the cell suspensions were incubated at 37°C. Lysis was monitored by following the decrease in OD_600_ of the samples at 15-min intervals for 90 min, as previously described [41]. The presented results are the averages from three independent experiments, each performed in triplicate.

### HPLC analysis of the muropeptide composition of peptidoglycan

The peptidoglycan of the studied strains was isolated, purified and then digested with the muramidase mutanolysin M1 (Sigma-Aldrich) as described previously [42], except that the purified murein was not N-acetylated before digestion. Soluble muropeptides were reduced by treatment with sodium borohydride and then analyzed by HPLC on a Hypersil octadecylsilane (ODS) reversed-phase column (Teknochroma) according to the method of Glauner [43]. Eluted compounds were detected by monitoring the A_205_. HPLC analysis was performed twice using samples prepared in two independent peptidoglycan isolations.

### Estimation of teichoic acids content

The TA content in the cell walls of the studied strains was determined on the basis of the cell wall phosphate concentration, essentially as described previously [41]. Briefly, *L. monocytogenes* cultures were harvested by centrifugation and each cell pellet resuspended in 50 mM Tris-HCl (pH 7.5), then sonicated to disrupt the cells. Any unbroken cells were pelleted by centrifugation (7,000 × g, 10 min, 4°C). The supernatant was removed and mixed with an equal volume of hot 8% sodium dodecyl sulfate (SDS), then boiled for 30 min. The insoluble cell wall preparation was collected by centrifugation (150,000 × g, 30 min) and washed five times with distilled water. The SDS-free cell walls were then mineralized and the phosphate concentration determined according to the method of Chen [44]. The presented results are the averages from three independent experiments, i.e. performed using three separate cell wall isolations, each carried out in triplicate.

### Construction of *lacZ* transcriptional fusions and β-galactosidase assay

DNA fragments containing the promoter regions of genes of interest were amplified by PCR using the primer pairs listed in Table 1. The fragments were digested with *Eco*RI and *Bam*HI (except that representing the *dlt* operon promoter, which was digested with SmaI and *Bam*HI) and cloned into plasmid pTCV-*lac* [45], a shuttle vector for the construction of transcriptional fusions with the *lac*Z gene, digested with the corresponding restriction enzymes. Correct insertion of the promoter fragments into pTCV-*lac* was verified by DNA sequencing and the resulting plasmid constructs were introduced into the wild-type and Δ*fri* mutant strains by electroporation [46]. For measurement of *lac*Z expression, cells from the *L. monocytogenes* cultures were collected by centrifugation and assayed for β*-*galactosidase activity [47]. Briefly, the cells were permeabilized by treatment with 0.5% toluene/4.5% ethanol, and the enzyme activity was determined according to a standard protocol [48]. The specific activity of β-galactosidase was calculated using the formula 10^3^ × (OD_420_ of the reaction mixture - OD_550_ of the reaction mixture)/(reaction time in minutes × OD_600_ of the cells used in the reaction mixture). The specific β-galactosidase activities presented are the averages from three independent experiments, each performed in triplicate.

**Table 1:** Oligonucleotide primers used in this study.

Primer	Sequence (5′ → 3′)
auto F^a^	GA**GAATTC**AGTAGATAAACGTACCGATTGGA
auto R b	GA**GGATCC**GCTGCTTTAGCATGGAATAGAG
ami F^a^	CT**GAATTC**CGCTGCCGGATTGTATATTA
ami R b	CT**GGATCC**TAATCATAGTAGCGGAGGTGC
iap F^a^	TT**GAATTC**GCGGGCTATTTCTCGATACTG
iap R b	TT**GGATCC**TGTAGCCGCGATAGTTGCT
murA F^a^	TA**GAATTC**GCCATCATACAAAGTGTTGC
murA R b	TA**GGATCC**ACCCCAGCAATTGTTGCACC
spl F^a^	TC**GAATTC**GAAGTTAGTGACAAGCTCGC
spl R b	TC**GGATCC**TGCTGCGAGTGAGATCGC
ftsEX-spl F^a^	GT**GAATTC**GTGCAAGTGAAGTAATGGGTGA
ftsEX-spl R b	GT**GGATCC**GGCAGCTGTTATGCCGTTAG
dltABCD F^c^	GCG**CCCGGG**ACAAGCCTTTTCTCCTGCA
dltABCD R^b^	CG**GGATCC**AGGGAAATCCGGTGTCTTCT
secA2 F^a^	TC**GAATTC**TGTAGCCGCGATAGTTGCT
secA2 R b	TA**GGATCC**GCGGGCTATTTCTCGATACTG

a The sequence in boldface type is the EcoRI restriction enzyme site.

b The sequence in boldface type is the BamHI restriction enzyme site.

c The sequence in boldface type is the SmaI restriction enzyme site.

### Protein isolation

Protein isolation was performed according to a previously described cell fractionation method [49] with modifications regarding the isolation of supernatant and cell wall proteins. Briefly, *L. monocytogenes* cultures were harvested by centrifugation and secreted proteins were isolated from the culture supernatants by precipitation with trichloroacetic acid (at a final concentration of 10%) as described previously [50]. The cell pellets were resuspended in phosphate-buffered saline (PBS) and cells disrupted by sonication. Following the removal of unbroken cells by centrifugation (7,000 × g, 10 min, 4°C), the cytoplasm was separated from the cell envelope by centrifugation (100,000 × g, 45 min). The supernatants containing the cytoplasmic proteins were collected and the pellets were resuspended in 0.5% sodium sarcosinate in PBS. The mixtures were then incubated at 25°C for 30 min with shaking. After centrifugation (100,000 × g, 45 min), the supernatants containing the cytoplasmic membrane proteins were collected. The pellets containing the peptidoglycan layer of the cell wall with noncovalently associated murein hydrolases [51] were resuspended in sample buffer (1% SDS, 5% 2-mercaptoethanol) and the cell wall-associated proteins were released by boiling the samples. The isolated proteins from various cell fractions were adjusted to a final volume that was commensurate with the cell density (0.20 ml/10^10^ cells).

### Protein analysis by SDS-PAGE and Zymography

Isolated protein fractions were analyzed by sodium dodecyl sulfate (SDS) - 10% polyacrylamide gel electrophoresis (PAGE). After electrophoresis the gels were stained with Coomassie brilliant blue to visualize protein bands. Murein hydrolases were analyzed by renaturing gel electrophoresis as described previously [52], using 8–12% SDS-polyacrylamide gradient gels containing 0.1% lyophilized *Micrococcus luteus* cells (Sigma-Aldrich). After electrophoresis the gels were washed with 25 mM Tris-HCl (pH 7.5) containing 1% Triton X-100 and incubated with shaking at 37°C for 24–48 h. Bands of murolytic activity were detected by staining with 1% methylene blue (Sigma) in 0.01% KOH and subsequent destaining with distilled water. Murein hydrolase activity was visualized as zones of clearing in the blue-stained cell wall background, which were analyzed by densitometry using an ImageQuant™ 300 and ImageQuant™ TL software (GE Healthcare, United Kingdom). SDS-PAGE and zymography analysis were performed three times using proteins from three independent isolations.

### Preparation of samples for electron microscopy

Bacterial cells were prepared for observation under a scanning electron microscope (SEM) as described previously [53]. Briefly, *L. monocytogenes* cultures were harvested by centrifugation and the cells were fixed by incubating for 30 min in 4% paraformaldehyde. After washing three times in PBS (pH 7.4), the cells were dehydrated using a graded ethanol series (25%, 50%, 75%, 96% ethanol; 15 min for each step). The cell suspension was then spread on a microcover, coated with gold and examined using a LEO 1430VP scanning electron microscope. For observation under a transmission electron microscope (TEM), the cells were prepared by thin sectioning as described previously [54]. Briefly, the cells were fixed (12 h, RT) in 1.5% glutaraldehyde in 0.1 M cacodylate buffer (pH 7.2). After washing three times in 0.1 M cacodylate buffer the cells were resuspended in cacodylate buffer and incubated for 30 min at RT. This step was repeated 4 times prior to fixing the cells in OsO_4_ solution for 2 h. The cell suspension was then dehydrated using a graded ethanol series (70%, 80%, 96%, absolute ethanol) followed by acetone. The dehydrated material was embedded in Epon 812 and thin sections were prepared using Tesla microtome. The samples were examined using a Leo 1912AB transmission electron microscope. Samples for electron microscopy were prepared twice for each strain using two independent bacterial cultures.

### Statistical analysis

Statistical analysis was performed using STATISTICA® v10.0. Data were analyzed by Student's t-test, with differences considered significant when *P* values were □ 0.05.

## Results and Discussion

### Cell wall integrity of *L. monocytogenes* EGD and Δ*fri* mutant strains

**Figure 1:**
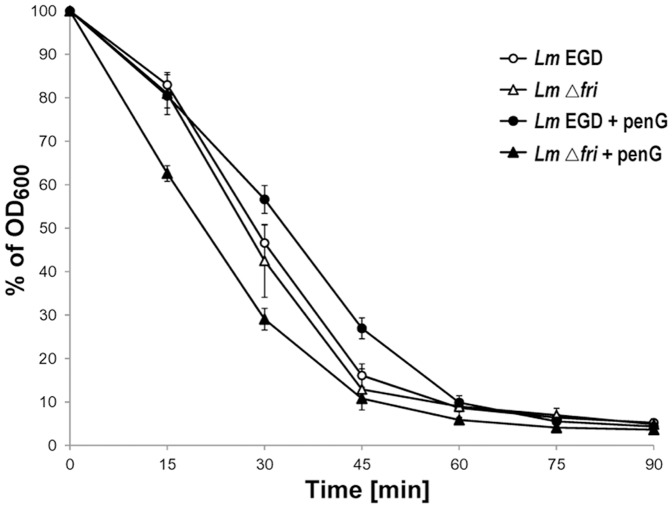
Integrity of the cell wall of *L. monocytogenes* strains. Cells of the wild-type (*Lm* EGD) and *Δ* *fri* mutant (*Lm* *Δ* *fri*) strains grown in the absence or presence of penicillin G (+penG) were incubated with lysozyme (10 U/ml) at 37°C for 90 min. At selected intervals, the OD_600_ of the cell suspensions was determined. Error bars represent the standard deviation from three independent experiments, each performed in triplicate.

It has recently been shown that the Fri protein of *L. monocytogenes* plays a crucial role in cell death caused by β-lactam antibiotics [20]. Detailed studies were undertaken to define the involvement of Fri in the maintenance of *L. monocytogenes* cell envelope structure and stability under β-lactam pressure. In this study, we compared the properties of wild-type strain EGD and the *Δ* *fri* mutant grown in both the absence and presence of 0.09 µg/ml of penicillin G (corresponds to 0.75 of the MIC value). These β-lactam stress conditions were previously shown to permit efficient growth of both strains, but caused slight growth retardation of strain *Δ* *fri* compared to the wild type [20]. To examine the role of Fri in maintaining the integrity of the *L. monocytogenes* cell envelope under β-lactam pressure, the susceptibility of the studied strains to lysis induced by incubation with the cell wall hydrolase lysozyme was assessed (Figure 1). When strains grown without antibiotic pressure were used in this experiment, the rate of cell lysis of the *Δ* *fri* mutant was not significantly different to that observed for the wild-type strain. However, when the strains grown under penicillin G pressure were compared, the rate of cell lysis of the mutant strain was significantly increased. In contrast, wild-type strain EGD was less susceptible to the action of lysozyme than both the mutant strain grown under the same conditions and EGD grown without the antibiotic (Figure 1). This result demonstrates that in response to penicillin G pressure, cells of *L. monocytogenes* become less susceptible to lysis and that the lack of *fri* expression in these adverse conditions leads to impaired cell wall integrity. Subsequently, we examined whether the observed increase in the susceptibility of *Δ* *fri* cells to lysozyme was an effect of changes in the level of N-deacetylation of peptidoglycan, as has been observed in the case of the *pdgA* mutant of *L. monocytogenes* [55], [56]. However, HPLC analysis did not reveal any significant differences in the muropeptide composition of peptidoglycan of either strain, grown with or without penicillin G (Figure S1). Thus, the observed decrease in the integrity of the cell envelope of the *Δ* *fri* mutant was not due to alteration of the structure of the main component of the cell wall, i.e. peptidoglycan.

### Role of Fri in teichoic acids synthesis

In a further attempt to explain the reduced integrity of the cell envelope of the *Δ* *fri* strain grown under β-lactam pressure, we examined the role of Fri in teichoic acid synthesis. Teichoic acids, along with peptidoglycan, are the main components of the cell wall of Gram-positive bacteria and are thought to control the overall surface charge and affect murein hydrolase activity [17]. *L. monocytogenes* mutants in genes involved in the synthesis of teichoic acids exhibit decreased integrity of the cell envelope [41], [42]. Furthermore, teichoic acids have been shown to contribute to lysozyme resistance in staphylococci, by preventing the binding of this cell wall hydrolase to its peptidoglycan target [57]. Examination of the TA content in the cell wall of the *Δ* *fri* mutant and wild-type strains during growth without antibiotic pressure revealed similar levels of cell wall phosphate (Table 2). In wild-type cells grown in the presence of penicillin G, the amount of cell wall phosphate increased significantly, but no such increase was observed in the case of the mutant strain (Table 2). This result indicates that in response to β-lactam pressure, the content of teichoic acids in the cell wall of *L. monocytogenes* increases in a process that depends on the presence of functional Fri protein.

**Table 2:** Cell wall TA content in *L. monocytogenes* strains.

Strain	Mean cell wall phosphate (µmol/mg of cell wall) ± SD^a^	
	BHI medium	BHI medium + PenG
**EGD**	1.47 ± 0.04	1.79 ± 0.03 b
**Δ** ***fri***	1.54 ± 0.05	1.41 ± 0.04^c^

a The results are the average of three independent experiments, each performed with separate cell wall preparations of the strains grown in the presence (+ PenG) or absence of 0.09 µg/ml penicillin G ± the standard deviation (SD).

b Significant differences following growth in the presence and absence of penicillin G (Student's t-test; P<0.05).

c Significant differences between the studied strains (Student's t-test; P<0.05).

### Morphology of *L. monocytogenes* EGD and Δ*fri* mutant strains

Abnormal cell wall structure and cell morphology have been observed in *L. monocytogenes* when the content of teichoic acids in the cell wall is reduced [41], [42]. In daptomycin-resistant isolates of *S. aureus* an increase in the TA content was accompanied by an increase in the thickness of the cell wall [58]. Thus, it may be concluded that changes in teichoic acids content often result in alterations in the structure of the Gram-positive cell wall, which can in turn influence cell morphology. Given the observed involvement of Fri in the control of cell envelope integrity as well as its influence on the content of teichoic acids in the cell wall of *L. monocytogenes* under β-lactam pressure, the effect of the *fri* mutation on cell morphology and the structure of the cell wall was examined.

**Figure 2:**
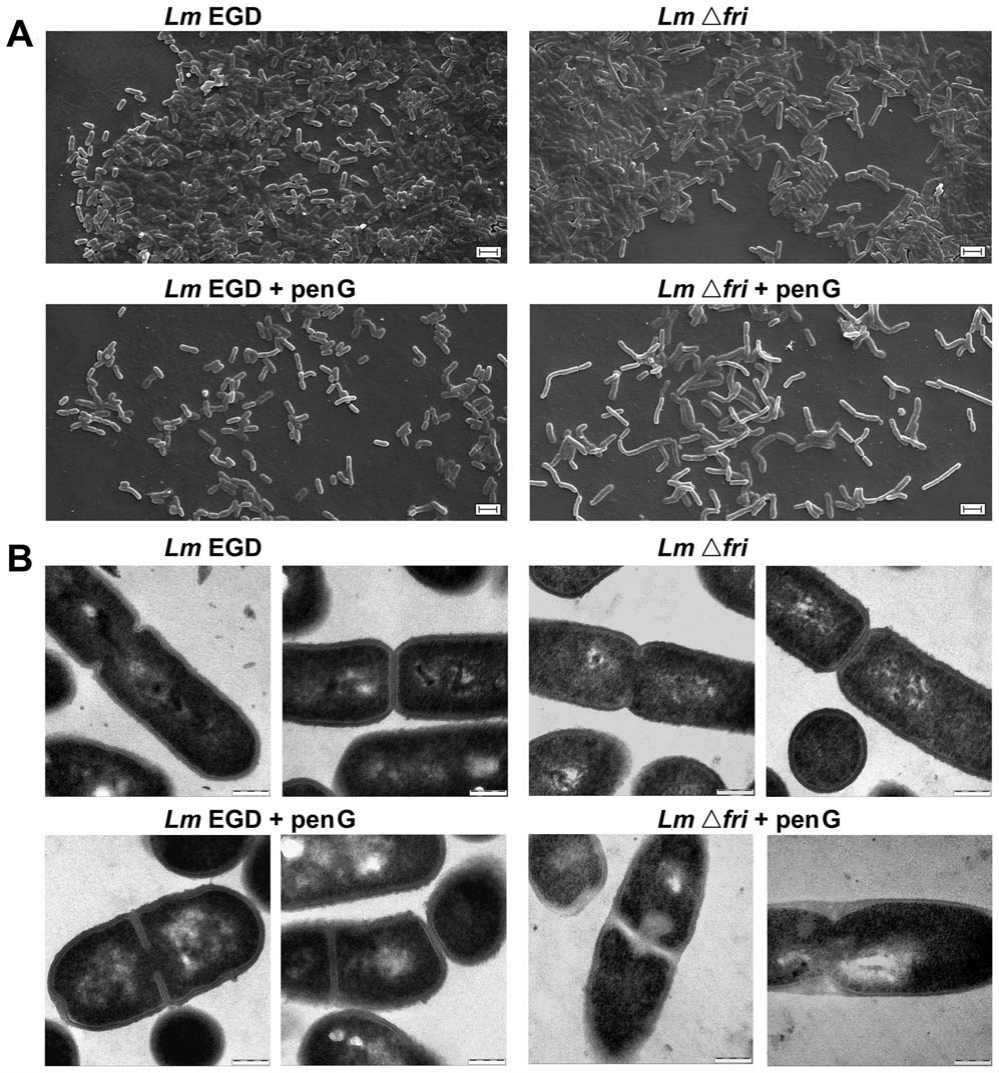
Cell morphology of *L. monocytogenes* strains. Scanning electron micrographs (A) and transmission electron micrographs (B) of wild-type (*Lm* EGD) and *Δ* *fri* mutant (*Lm* *Δ* *fri*) cells grown in the absence or presence of penicillin G (+penG). Bars indicate 2 µm in the scanning electron micrographs and 200 nm in the transmission electron micrographs. The presented micrographs are representative of two independent sample preparations.

Scanning electron microscopy (SEM) revealed that cells of the *L. monocytogenes* strain lacking *fri* were significantly longer than those of the wild type, in both the absence and presence of penicillin G (Figure 2A). However, this effect was much greater in the latter conditions (Table 3) and there was also an increased percentage of *Δ* *fri* cells with irregular morphology in the presence of β-lactam pressure. In contrast, cells of the wild-type EGD strain were significantly shorter following growth in the presence of penicillin G with an increased percentage of cells linked in chains.

**Table 3:** Cell length and cell wall thickness of *L. monocytogenes* strains.

Strain	Average cell length (µm) ± SD^a^		Average cell wall thickness (nm) ± SD^b^	
	BHI medium	BHI medium + PenG	BHI medium	BHI medium + PenG
**EGD**	1.52 ± 0.25	1.35 ± 0.22^c^	26.52 ± 1.82	30.78 ± 2.86^c^
**Δ** ***fri***	2.02 ± 0.39^d^	2.37 ± 0.76c,d	25.59 ± 1.67	18.81 ± 2.49c,d

a Mean cell lengths were determined by measuring 100 cells of each strain grown in the presence (+ PenG) or absence of 0.09 µg/ml penicillin G. The analysis was performed using two independently prepared samples. From each independent sample, equal numbers of cells were analyzed ± standard deviation (SD).

b Mean cell wall thickness was determined by measurement of 20 cells of each strain grown in the presence (+ PenG) or absence of 0.09 µg/ml penicillin G. The analysis was performed using two independently prepared samples. From each independent sample, equal numbers of cells were analyzed ± standard deviation (SD).

c Significant differences following growth in the presence and absence of penicillin G (Student's t-test; P<0.05).

d Significant differences between the studied strains (Student's t-test; P<0.05).

A detailed insight into the structure of the cell wall of the studied strains was obtained using transmission electron microscopy (TEM) (Figure 2B). In thin sections, both wild-type and *Δ* *fri* cells grown in the absence of the antibiotic presented the typical morphology of dividing bacteria, with the formation of a regular septum and normal cell wall thickness, which did not vary in the studied strains. In the presence of penicillin G, strain EGD had a normal morphology and no disorders in septum formation were observed, but the cell wall was significantly thicker (Table 3). In contrast, the *Δ* *fri* mutant grown in the presence of this β-lactam, exhibited a clearly altered morphology with reduced cell wall thickness, frequently accompanied by deformities. Furthermore, cell division was apparently arrested in many cells, with irregularities observed in septum structure. Together, these data indicate that *fri* expression is required to maintain both the shape of the bacterial envelope and normal cell division during the growth of *L. monocytogenes* under penicillin G pressure.

### Participation of Fri in regulating the transcription of murein hydrolase genes and genes engaged in controlling the activity of murein hydrolases

Morphological changes, like the inability of daughter cells to separate following cell division, are the most frequently observed effect of murein hydrolase gene mutations in both Gram-positive and Gram-negative bacteria [16]. Likewise, cell division defects are clearly visible in *L. monocytogenes* strains deficient in two cell-wall hydrolases, Iap and MurA [59], [60], [61], [62]. Taking into consideration the major influence of murein hydrolases on the integrity of the cell envelope and bacterial cell lysis, we decided to investigate the possible role of Fri in regulating the transcription of genes encoding the known murein hydrolases of *L. monocytogenes*, i.e. Auto, Ami, MurA, Iap and Spl (p45). The activity of murein hydrolases is often regulated at the posttranslational level, e.g. by controlling the secretion of these enzymes. It is noteworthy that in *L. monocytogenes*, the auxiliary secretion protein SecA2 was shown to be specifically engaged in the secretion of the murein hydrolases Iap and MurA [61], [62]. Therefore, we also investigated the involvement of Fri in the transcriptional regulation of the *secA2* gene. In many Gram-positive bacteria the *dlt* genes required for D-alanylation of teichoic acids play a vital role in modulating surface charge and controlling murein hydrolase activity [63], [64], [65]. Thus, the influence of Fri on the expression of the *dltABCD* operon was also examined.

To test whether some of the aforementioned genes are under the control of Fri, DNA fragments containing their putative promoter regions were amplified by PCR and fused to *lac*Z in the transcriptional fusion vector pTCV-*lac*. The resulting plasmids were introduced into the *L. monocytogenes* wild-type and *Δ* *fri* mutant strains, and the levels of β-galactosidase activity were determined. In the presence of penicillin G, the expression of the *iap-lac*Z, *murA-lac*Z and *secA2-lac*Z fusions was over 1.5-, 2.5- and 2-fold lower, respectively, than the expression under non-stress conditions in both strains (Table 4). This result demonstrates that the expression of these three genes is repressed by penicillin G only, and Fri is not involved in this regulation. Since secA2 is engaged in the secretion of Iap and MurA [61], [62], diminished membrane translocation of these two autolysins would be anticipated under β-lactam pressure. Interestingly, the expression of *dltABCD-lac*Zwas reduced 2-fold during growth with penicillin G, but only in the *Δ* *fri* strain. This indicates that expression of the *dlt* operon under β-lactam pressure is controlled by Fri.

**Table 4:** Expression of promoter-*lacZ* fusions in *L. monocytogenes* strains.

Promoter of gene or operon^b^	Expression^a^			
	BHI medium		BHI medium + PenG	
	EGD strain	Δ*fri* strain	EGD strain	Δ*fri* strain
*auto*	108.1 ± 8.4	97.4 ± 7.1	99.8 ± 19.3	102.9 ± 16.7
*ami*	85.1 ± 4.0	71.9 ± 3.6	80.2 ± 8.0	64.0 ± 10.2
*iap*	132.3 ± 9.0	128.2 ± 7.4	77.1 ± 8.4^c^	84.3 ± 6.3^c^
*murA*	145.7 ± 13.3	121.5 ± 11.8	52.6 ± 6.7^c^	45.8 ± 5.8^c^
*spl*	103.7 ± 5.3	96.9 ± 4.9	97.0 ± 7.1	103.7 ± 8.4
*ftsEX-spl* b	124.4 ± 12.4	115.4 ± 7.7	97.4 ± 5.9	95.2 ± 10.3
*dltABCD* b	114.4 ± 9.1	109.4 ± 6.2	101.2 ± 5.2	46.6 ± 4.3c, d
*secA2*	76.3 ± 9.5	67.5 ± 7.9	33.3 ± 6.8^c^	27.5 ± 8.5^c^

a The expression of promoter-*lacZ* fusions in response to the addition of 0.09 µg/ml penicillin G (+ PenG) or in the absence of the antibiotic was determined by β-galactosidase assays. Specific β-galactosidase activity was measured for wild-type (EGD) or Δ*fri* mutant cells containing promoter-*lacZ* fusions, grown in the presence or absence of the antibiotic. The results are the average of three independent experiments, each performed in triplicate ± standard deviation.

b Genes that are organized in an operon (according to Toledo-Arana et al. [66]).

c Significant differences following growth in the presence and absence of penicillin G (Student's t-test; P<0.05).

d Significant differences between the studied strains (Student's t-test; P<0.05).

### Influence of Fri on murein hydrolase content in various cellular compartments

Simple transcriptional analysis of murein hydrolase genes is not sufficient to estimate the activity of the encoded enzymes, since the regulation of murein hydrolase activity is highly complex and is often controlled at the posttranslational level [16]. As mentioned above, the activity of murein hydrolases can be controlled at the level of secretion. Furthermore, following membrane translocation, murein hydrolase activity can also be regulated by their stability and/or localization. There is only very limited information on the control of stability of *L. monocytogenes* autolysins. However, the proteolytic processing of autolysins seems to be common among Gram-positive bacteria, and has been observed in the case of MurA and Ami of *L. monocytogenes* [61]. The control of autolysin localization is also poorly understood. The expected final destination of murein hydrolases of *L. monocytogenes* is their site of action in the cell wall. These enzymes are noncovalently bound to the cell wall via their LysM domain or GW modules [51], [67]. However, autolysins with LysM and GW regions have been detected not only in the cell wall, but also in the supernatant fraction. Furthermore, Iap and Spl are also found in the cell membrane fraction [61], [68], [69], [70].

To determine the posttranslational fate of *L. monocytogenes* murein hydrolases and thus their potential activity, and to examine the effect of β-lactam pressure and/or Fri on the posttranslational regulation of these enzymes, we compared their abundance in different cell compartments of EGD and *Δ* *fri* strains grown with and without penicillin G. Proteins were isolated from each cellular fraction, i.e. cytoplasm, cytoplasmic membrane, cell wall and culture supernatant. SDS-PAGE analysis revealed that the protein patterns of each cellular fraction were similar in the mutant and the wild-type strains, irrespective of the presence of penicillin G (Figure S2). Therefore, the lack of Fri does not result in global alterations of protein composition or in the non-specific release of proteins into the culture supernatant.

**Figure 3:**
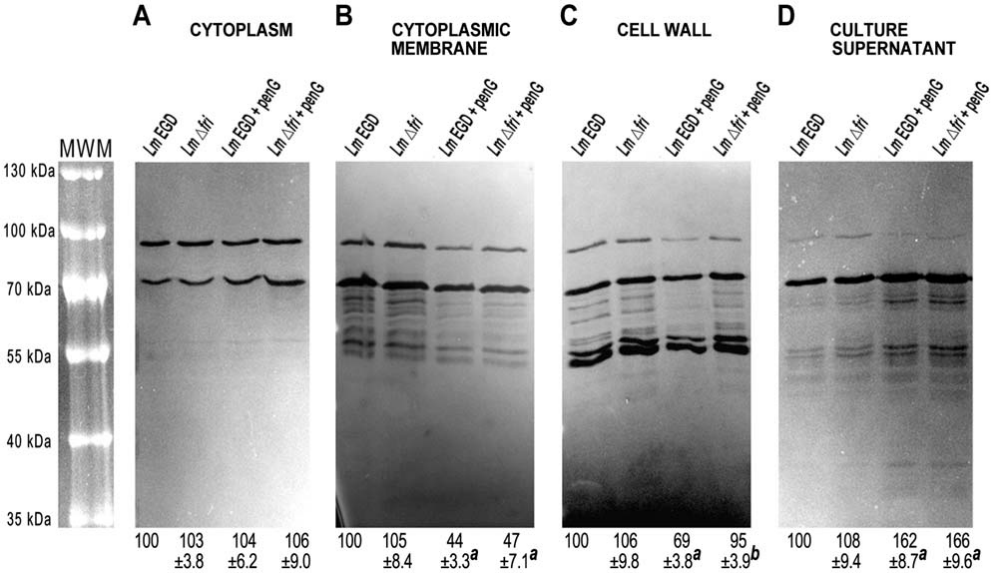
Content of murein hydrolases in various cellular compartments of *L. monocytogenes* strains. Zymography analysis of proteins from the cytoplasm (A), cytoplasmic membrane (B), cell wall fraction (C) and culture supernatant (D) were performed for the wild-type EGD strain grown without (*Lm* EGD) and with (*Lm* EGD + penG) penicillin G, and the *Δ* *fri* mutant strain grown without (*Lm* Δ*fri*) and with (*Lm* Δ*fri* + penG) the same antibiotic. Equivalent quantities of proteins isolated from each fraction were subjected to zymographic analysis in SDS-polyacrylamide gels. MWM – prestained Protein Molecular Weight Marker. The relative amounts of murein hydrolases estimated on the basis of the densitometric analysis are shown in the lower panel. Values in each analyzed compartment were normalized to the total intensity of murein hydrolases from the wild-type EGD strain grown without the antibiotic, which was assigned the value of 100%. The presented results are mean values from the analysis of three independent protein isolations ± the standard deviation. *^a^* Significant differences following growth in the presence and absence of penicillin G (Student's t-test; P<0.05). *^b^* Significant differences between the studied strains (Student's t-test; P<0.05).

After this preliminary assay, equivalent quantities from each fraction were subjected to zymography analysis (Figure 3A-3D). Densitometry of zymograms revealed that in the presence of penicillin G, the total amount of murein hydrolases recovered in the cell membrane fraction was approximately 55% lower than under non-stress conditions in the two strains (Figure 3B). Furthermore, in the presence of penicillin G, the total amount of murein hydrolases released into both culture supernatants was approximately 60% higher than under non-stress conditions (Figure 3D).

These results indicate that the diminished amount of cell membrane-associated murein hydrolases as well as the increased release of these enzymes into the culture supernatant are an effect of the presence of penicillin G alone. Fri is not involved in regulating the translocation of these enzymes across the membrane, nor in their release to the supernatant. Interestingly, the total amount of murein hydrolases recovered in the cell wall fraction of strain *Δ* *fri* was about 35% higher than in the wild-type strain during growth under penicillin G pressure, whereas during growth without the antibiotic there was no significant difference between the two strains in the level of these enzymes (Figure 3C). Furthermore, the amount of cell wall-associated murein hydrolases isolated from the wild-type strain was approximately 30% lower in cells grown under penicillin G pressure compared to those grown without this antibiotic. This reduction in the level of cell wall-associated murein hydrolases caused by penicillin G was not observed in the case of the *Δ* *fri* mutant strain (Figure 3C). The reduction in the amount of cell membrane- and cell wall-associated murein hydrolases in response to β-lactam pressure suggests that the content of these enzymes in these compartments is subject to tight regulation. The lack of downregulation of cell wall-associated murein hydrolases in the *Δ* *fri* strain clearly indicates that under these adverse conditions, Fri is indispensible in controlling the abundance of these enzymes in their site of activity.

The results of the cell wall integrity assay demonstrate that Fri is indispensable for the observed increase in the integrity of the *L. monocytogenes* cell wall in response to penicillin G pressure. The higher cell wall integrity is correlated with an increased content of teichoic acids in response to penicillin G pressure, which depends on the presence of the functional Fri protein. Furthermore, the results of transcriptional analysis revealed that expression of the *dlt* operon under penicillin G pressure is controlled by Fri, indicating that this protein is also involved in modulating the net charge of the teichoic acid polymers.

Interestingly, one of the most well characterized and widespread functions of teichoic acids among bacterial species is the control of autolysins [17], [18]. It has been proposed that teichoic acids might control autolysins directly since these enzymes have high affinities for these polyanionic polymers [71], [72]. It is also possible that the ability of teichoic acids to provide cations may be more important in controlling autolysins than direct interaction, since the activity of the major autolysins depends on bivalent cations [18], [73]. Alternatively, TA may play an indirect role in autolysin control by providing a proton-binding mechanism that regulates the enzyme activity by influencing acidification of the environment [74]. Furthermore, D-alanylation of teichoic acids is considered to be crucial in modulating surface charge and controlling murein hydrolase activity [63], [64], [65]. For example, *dlt* mutations in *B. subtilis* resulted in enhanced autolysis and the cell walls of the mutants were apparently more negatively charged [63], [75]. Thus, the observed decrease in the cell wall integrity of the *L. monocytogenes fri* mutant could result from insufficient control of autolysin activity, which might be the effect of a reduced content of TA that are poorly D-alanylated. Insufficient control of autolysin activity could lead to the reduced cell wall thickness and septum irregularities observed in the *fri* mutant under penicillin G pressure. With regard to the latter, it is noteworthy that in addition to being regulated by teichoic acids, autolysins may be positioned at cell wall septa by these polymers [71], [72].

As mentioned above, it has been proposed that D-alanylation determines the number of anionic sites on TA for autolysin binding. According to this hypothesis, strains with reduced *dlt* expression should bind more autolysin, resulting in an increased rate of autolysis [63]. In the light of this hypothesis, it seems likely that the observed higher content of autolysins in the *fri* mutant compared to the EGD wild type is the direct result of reduced D-alanylation in the mutant strain and the consequent higher binding capacity of the teichoic acids. Furthermore, the observed decrease in the cell wall integrity of the *fri* mutant might result both from its inability to regulate the autolysin content of the cell wall compartment as well as insufficient control of the activity of these enzymes. However, it should be stressed that the final amount of autolysins in the cell wall depends on processes such as their secretion, stability in the cell envelope and transport across the cell wall into the extracellular milieu. In this respect, D-alanylation could play another important role in modulating the rate of protein transport across the cell wall, since the net negative charge of the wall influences protein folding [76], [77].

The results of the present study show that a ferritin-like protein plays an important role in maintaining the stability and structure of the cell envelope of *L. monocytogenes* under β-lactam pressure. Previously, Fri was shown to be involved in protection against multiple stresses and the expression of the *fri* gene was found to be subject to a complex control mechanism involving Fur, Per and SigB [32], [33], [34], [37], [38]. Alternative sigma factor SigB was shown to determine the tolerance of *L. monocytogenes* to cell envelope-acting antimicrobial agents and is thought to control the transcription of genes contributing to this tolerance, including the *dlt* operon [78]. Interestingly, overexpression of an anti-sigma B factor, RsbW, was observed in a *fri* mutant strain, [37], which strongly suggests negative modulation of SigB activity by the Fri protein. This might explain the important role of Fri in maintaining the stability of the *L. monocytogenes* cell envelope observed in the current study. However, further studies are required to examine possible interactions between Fri and SigB under β-lactam pressure.

It is assumed that the important role of Fri in protecting the bacterial cells against multiple stresses relies on the prevention of oxidative damage by removing excess ferrous ions from the cytosol, making them unavailable for participation in the Fenton reaction. Interestingly, the study of Kohanski and co-workers revealed that bactericidal antibiotics increase cellular production of H_2_O_2_, the end product of an oxidative damage-cellular death pathway involving stimulation of the Fenton reaction [79]. This suggests that Dps proteins play a crucial role in the control of bacterial cell death caused by antibiotics. In line with this hypothesis is growing evidence of the role of Dps proteins in bacterial cell death in human pathogens including *Salmonella enterica*, *Mycobacterium tuberculosis* and *Listeria monocytogenes* [20], [80], [81]. Here, we describe for the first time, the effect of Dps protein on the stability and structure of the *L. monocytogenes* cell envelope under β-lactam antibiotic pressure. Under these adverse conditions the Dps protein plays an important role in regulating components that are critical for Gram-positive cell envelope function, i.e. the amount/activity of cell wall-associated murein hydrolases, the content of teichoic acids in the cell wall and the level of D-alanylation of teichoic acids.

## Conclusions

Serious illnesses and fatalities that occur in susceptible individuals highlight the importance of understanding the mechanisms underlying the innate tolerance of *L. monocytogenes* to β-lactam antibiotics. The findings of the present study demonstrate that Fri protein, which has recently been identified as a mediator of β-lactam tolerance, is involved in regulating *L. monocytogenes* cell envelope structure and integrity under β-lactam pressure. Our detailed investigation revealed the inability of an *L. monocytogenes* strain lacking Fri to control the amount and/or activity of cell wall-associated murein hydrolases. This deficiency is caused by the inability of the *Δ* *fri* strain to (i) reduce the content of these enzymes associated with the cell wall, (ii) adjust the content of teichoic acids bound within the cell wall and (iii) maintain the D-alanylation of teichoic acids at the proper level under β-lactam pressure. We provide evidence that under β-lactam pressure, the lack of a functional Fri protein leads to the loss of control of both the amount and activity of cell wall-associated murein hydrolases, which has serious implications for *L. monocytogenes* cell envelope structure and stability in these conditions.

Furthermore, the results of the present study demonstrate that the content of cell wall-associated murein hydrolases in *L. monocytogenes* is subject to tight control in response to β-lactam pressure, which provides insight into the mechanism of *L. monocytogenes* tolerance to this class of antibiotics. The observed fate of murein hydrolases in *L. monocytogenes* cells grown under β-lactam pressure indicates the presence of a multilevel mechanism controlling murein hydrolase activity. This mechanism includes transcriptional downregulation of genes encoding the two main murein hydrolases and SecA2 protein involved in their secretion. Moreover, an analysis of the abundance of murein hydrolases in different cell compartments showed that penicillin G treatment causes a decrease in the relative content of these enzymes associated with the cell membrane and cell wall, and an increase in the relative content of these enzymes released into the supernatant. In light of these observations, we propose that the alterations in the level and/or activity of cell wall-associated murein hydrolases, accompanied by an increase in the amount of TA in this compartment, led to the observed increase in cell wall thickness and cell envelope integrity of *L. monocytogenes* grown in the presence of penicillin G. Undoubtedly, these changes help to protect the cells from lysis and provide the basis for the innate tolerance of *L. monocytogenes* to β-lactams.

## Supporting Information

Figure S1 **HPLC analysis of the muropeptide composition of the peptidoglycan of** ***L. monocytogenes*** **strains.** The analyzed peptidoglycan was purified from the wild-type EGD strain (*Lm* EGD) and the *Δ* *fri* mutant strain (*Lm* Δ*fri*) grown without the antibiotic (A), and the wild-type EGD strain (*Lm* EGD + penG) and the Δ*fri* mutant strain (*Lm* Δ*fri* + penG) grown in the presence of penicillin G (B). Muropeptides produced by the enzymatic hydrolysis of peptidoglycan were reduced and separated by reversed-phase HPLC and the A_205_ of the eluate was monitored. The presented results are representative of HPLC analysis of two independent peptidoglycan preparations.
(TIF)

Figure S2 **Analysis of proteins isolated from different cellular compartments of** ***L. monocytogenes*** **strains.** Equivalent quantities of protein from the cytoplasm (A), cytoplasmic membrane (B), cell wall fraction (C) and culture supernatant (D) were subjected to SDS-PAGE analysis and stained with Coomassie brilliant blue. This analysis was performed for proteins isolated from cells of the wild-type EGD strain grown without (*Lm* EGD) and with (*Lm* EGD + penG) penicillin G, and the Δ*fri* mutant strain grown without (*Lm* Δ*fri*) and with (*Lm* Δ*fri* + penG) this antibiotic. MWM – prestained Protein Molecular Weight Marker. The presented results are representative of the analysis of three independent protein preparations.
(TIF)
