# Supplementary figures and images for: Critical Role of a Ferritin-Like Protein in the Control of Listeria monocytogenes Cell Envelope Structure and Stability under β-lactam Pressure
Source: PLoS One. 2013 Oct 24;8(10):e77808. doi: 10.1371/journal.pone.0077808 (PMC3812014; doi:10.1371/journal.pone.0077808)

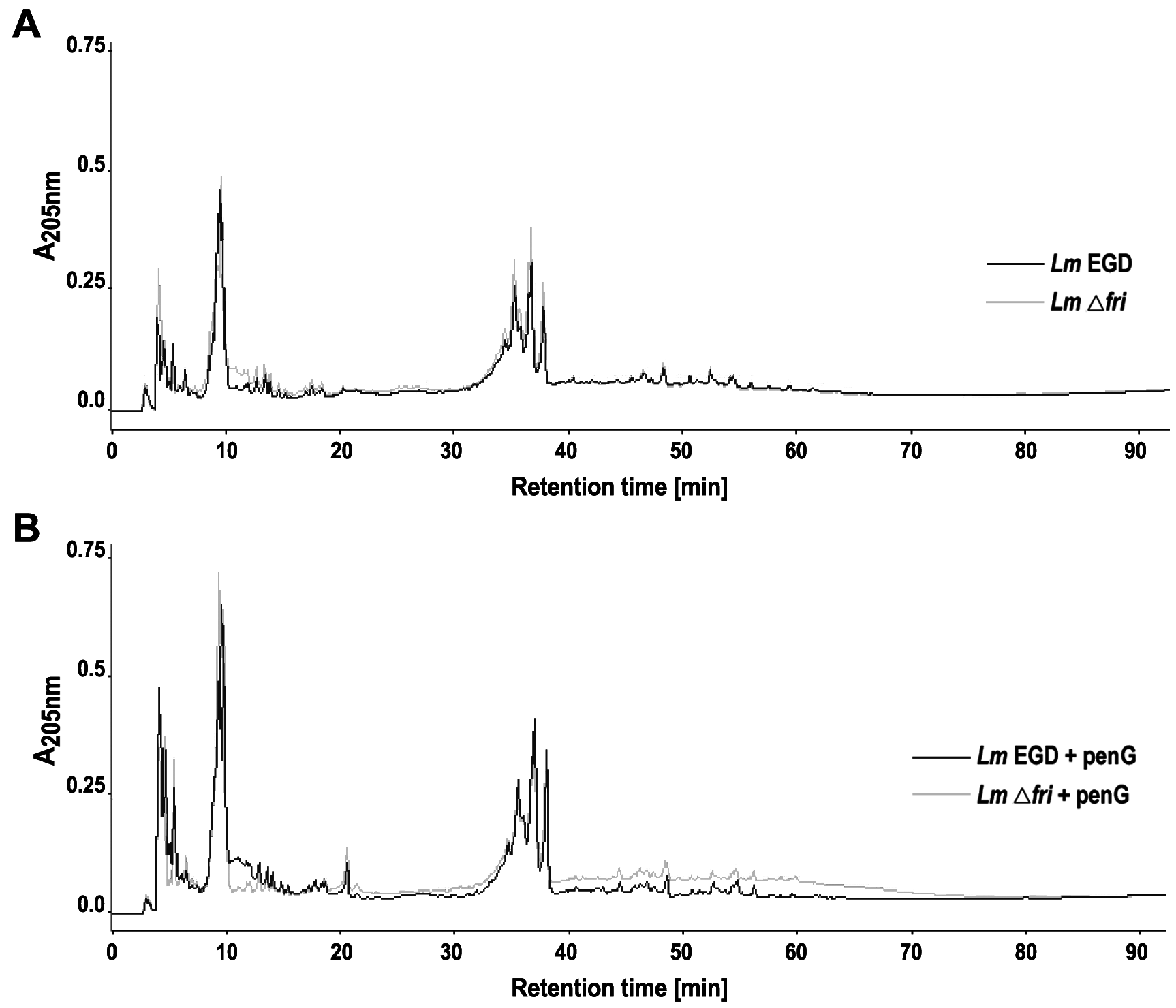

Supplement: Figure S1 — HPLC analysis of the muropeptide composition of the peptidoglycan of L. monocytogenes strains. The analyzed peptidoglycan was purified from the wild-type EGD strain (Lm EGD) and the Δ fri mutant strain (Lm Δfri) grown without the antibiotic (A), and the wild-type EGD strain (Lm EGD + penG) and the Δfri mutant strain (Lm Δfri + penG) grown in the presence of penicillin G (B). Muropeptides produced by the enzymatic hydrolysis of peptidoglycan were reduced and separated by reversed-phase HPLC and the A205 of the eluate was monitored. The presented results are representative of HPLC analysis of two independent peptidoglycan preparations. (TIF) [file pone.0077808.s001.tif]

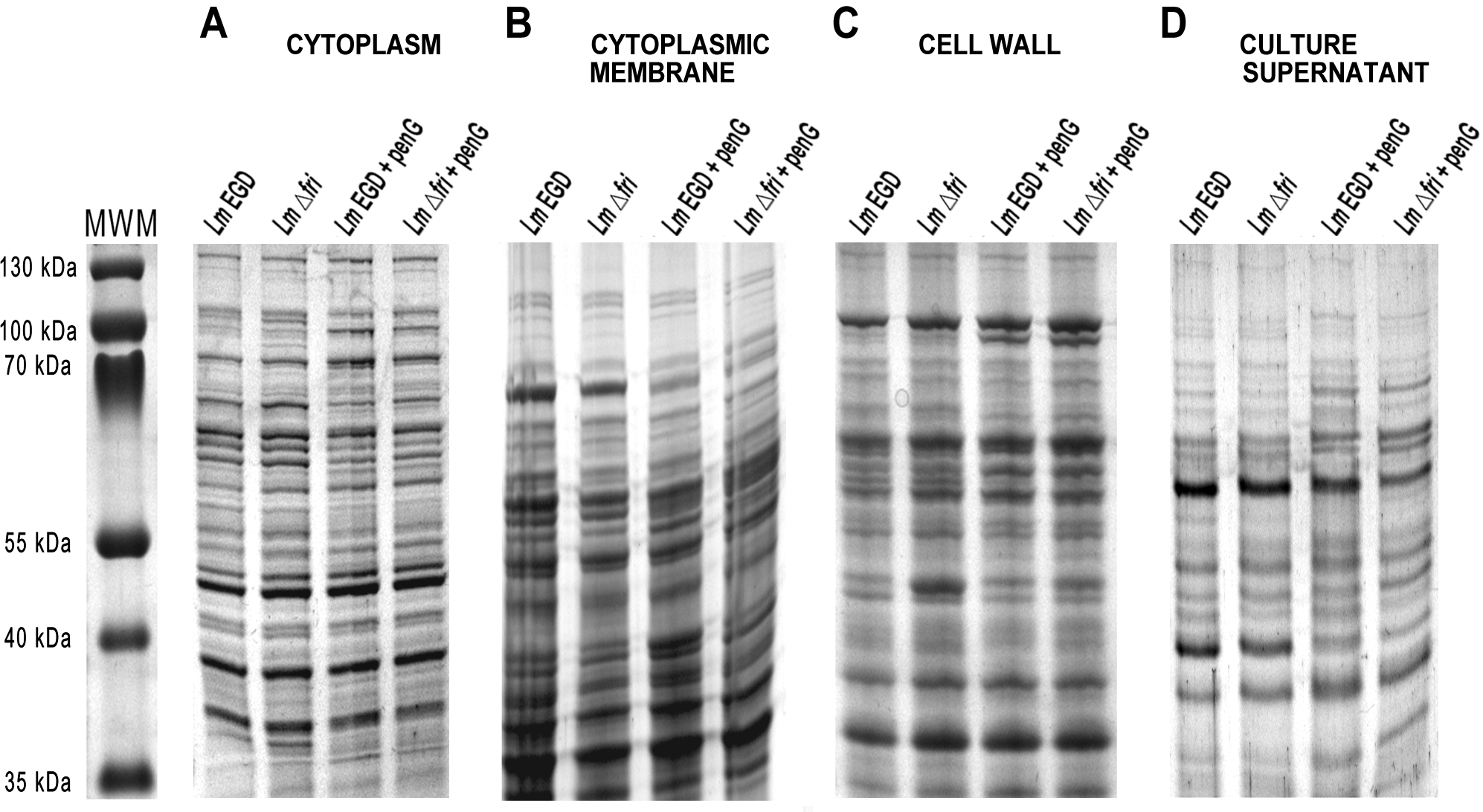

Supplement: Figure S2 — Analysis of proteins isolated from different cellular compartments of L. monocytogenes strains. Equivalent quantities of protein from the cytoplasm (A), cytoplasmic membrane (B), cell wall fraction (C) and culture supernatant (D) were subjected to SDS-PAGE analysis and stained with Coomassie brilliant blue. This analysis was performed for proteins isolated from cells of the wild-type EGD strain grown without (Lm EGD) and with (Lm EGD + penG) penicillin G, and the Δfri mutant strain grown without (Lm Δfri) and with (Lm Δfri + penG) this antibiotic. MWM – prestained Protein Molecular Weight Marker. The presented results are representative of the analysis of three independent protein preparations. (TIF) [file pone.0077808.s002.tif]
